# Socioeconomic factors influencing knowledge and consumption of food plants by a human group in a mountainous environment in the semiarid region of Bahia, Northeast Brazil

**DOI:** 10.1186/s13002-022-00542-8

**Published:** 2022-06-15

**Authors:** Luciana Vitor da Silva Souza, Juracy Marques, Letícia Zenóbia de Oliveira Campos, Ernani Machado de Freitas Lins Neto

**Affiliations:** 1 Programa de Pós-Graduação em Ecologia Humana e Gestão Socioambiental, Universidade do Estado da Bahia-UNEB, Juazeiro, Brazil; 2 Programa de Pós-Graduação em Ciências da Saúde e Biológicas, Universidade Federal do Vale do São Francisco-UNIVASF, Petrolina, Brazil; 3 Universidade Federal do Oeste da Bahia (UFOB), Barreias, Brazil

**Keywords:** Edible plants, Semiarid, Caatinga, Mountainous environment, Ethnobotany

## Abstract

**Background:**

The relationship of people with natural resources is guided by different sociocultural, ecological and evolutionary factors. Regarding food plants, it is not different. Studies around the world have evaluated the effects of socioeconomic factors, such as age, gender, income, profession, education level, time of residence, ethnic diversity, religion, festive rituals, access to urban areas and migrations. In this sense, the objective of the present study was to characterize the diversity of knowledge and use of food plants by people from Serra dos Morgados and evaluate if the socioeconomic factors influence knowledge and consumption of food plants in the community.

**Methodology:**

This research was conducted in the village of Serra dos Morgados, municipality of Jaguarari, Bahia, with the purpose of evaluating the factors that influence in the knowledge and use of food plants. Socioeconomic data such as age, gender, time of residence, and monthly income were collected. The free list technique was applied during the collection of ethnobotanical data in order to analyze the preference of the plants based on the salience index (SI). To analyze the factors that influence knowledge and use forms, we used GLM Lasso.

**Results:**

A total of 33 people were interviewed, 8 men and 25 women; their age ranged from 30 to 82 years. People cited 98 species of plants, 41 species being identified of spontaneous occurrence. The plant with the highest salience index (SI) was “cheirosa” (*Psidium ganevii)* (SI = 0.5679), followed by “massaranduba” (*Micropholis sp.*) (SI = 0.4323); “araça” (*Campomanesia guazumifolia*) (SI = 0.3320); and “cambuí” (*Siphoneugena sp.*) (SI = 0.3144).

**Conclusions:**

The main factors that influence knowledge and use forms in the locality were family income and the collection site, with homegardens cited as the preferred area for collection of food plants. This study provided an overview related to potentially important species for a community located in a region where there are few ethnobiological studies. The results presented here can be used in future studies, providing clues for investigations. Also, there is a contribution to the conservation of biocultural aspects related to the use of food plants in a community living in mountainous regions.

## Background

The relationship between people and food plants is influenced by many socioeconomic factors such as age, gender, income, education level, time of residence, religion, access to urban centers and migrations [1–5]. As an example, we can bring a study carried out in West Africa, Benin region, in which the authors concluded that different socioeconomic contexts shaped the diversity of food species used by populations [3]. In this same study, it was evidenced that ecological aspects related to the aridity of the regions also influenced the use of food species [3].

In addition to these variables, research carried out in different regions of the world observed that the availability, the abundance of resources and the species richness also influence the use of food plants [4, 6, 7]. Despite an increased interest in the study of food plants, much remains to be known about the diversity of use in this cultural domain, especially in the northeast of Brazil [8]. In this sense, we understand that obtaining a deep knowledge of biodiversity can be an important ally to propose alternatives that help improve the food security of people living in certain regions of the world, especially in regions that have been suffering the impacts caused by climate change, as well as other social problems.

However, there are still gaps that make it difficult to understand how certain socioeconomic and ecological contexts shape the use of biodiversity. Therefore, improving understanding of the four key dimensions behind food security: availability, utilization, accessibility and stability of a food system, is important.

Despite an increase in research that analysis of socioecological factors involved in the selection and use of plant resources, it is still necessary to investigate, in detail, the influence of certain variables to the detriment of others. For example, Sansanelli et al. [10] only studied the influence of gender on food plant use, while Çakir [11] evaluated the influence of age, more specifically the maintenance of knowledge and use of food resources throughout age-groups. Although the interest of researchers around the world in carrying out research on the influence of socioecological variables on the knowledge and use of food resources has been growing, the present study is important, as it expands this debate to the mountain environment, in which few studies are found [12]. These environments have a wide variety of complex and interrelated ecological systems [13]. They are mosaics of plant biodiversity that foster the development of much knowledge about natural resources [14]. This makes it interesting to conduct studies about the dynamics of knowledge and use of these natural resources. There has, for example, been a study carried out with women on food in mountainous regions of India, in which many species that have important cultural value were found. In this same study, it was found that these ecosystems contribute significantly to the conservation and use of biodiversity [15].

So, the objective of the present study was twofold: (i) to characterize the diversity of knowledge and use of food plants by people from Serra dos Morgados, highlighting the methods of preparation, preferential places for the collection and sale of food plants; and (ii) to evaluate if the socioeconomic factors that influence knowledge are similar to those that influence the consumption of food plants in the community. In this way, the following hypotheses were raised: (i) the interviewees significantly use the species they know, preferring to collect those plants closest to their homes and that add greater economic value; (ii) in the Serra dos Morgados community, women know and use more food species than men; in addition, older people tend to know more species, as well as those who have lived longer in the community.

## Methods

### Research area

Research was conducted in the community of Serra dos Morgados, municipality of Jaguarari, Bahia, Brazil. The municipality of Jaguarari present the estimated population of 33,746 inhabitants and an area of 2.466,009 Km^2^ based on data collected in 2020 [16]. The demographic density was 12,35 Hab/Km^2^ and the MHDI—Municipal Human Development Index—was 0.659 [16]. The climatic type of the municipality of Jaguarari is semiarid and susceptible to prolonged periods of drought or water scarcity.

As part of the “Cordillera do Espinhaço,” with an extension of 6000 to 7000 km^2^, the village “Serra dos Morgados (SM)” is located in a humid/sub-humid enclave of the “Serras da Jacobina,” today called “Serras do Sertão” North of Bahia, belonging to the municipality of Jaguarari. Also known as “Serra de Baixo,” in comparison with the neighboring community, “Serra de Cima” (“Berinjela” community), the SM has vegetation cover with patches of Caatinga, Cerrado and, above all, Atlantic Forest.

**Fig. 1:**
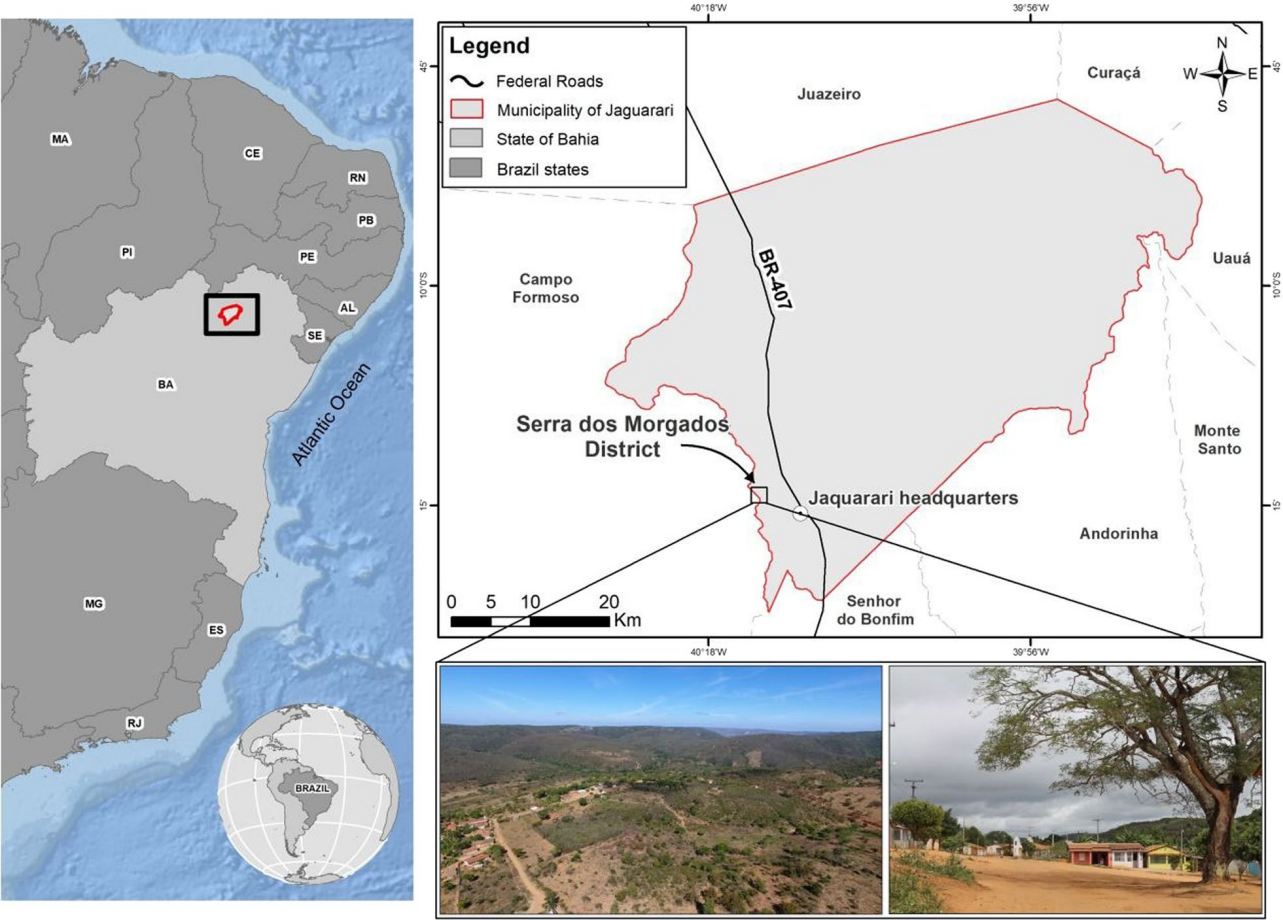
Map of community Serra dos Morgados, Jaguarari-Bahia 2020. Authors

The village of Serra dos Morgados (Fig. 1), also known as Serra de Baixo, is located 9 km from of the municipality of Jaguarari, at coordinates 10° 14′ 18.7 S and 40° 14′ 31.2 W, and an approximate altitude of 980 m. There are two associations in the village, one is for residents of Serra dos Morgados with 86 members and the other is exclusively for women. There is a healthcare unit with one healthcare worker. There is an elementary school (first to fifth grades) that has two teachers. The community was chosen due to some aspects detailed below: Other studies are already taking place in the locality, thus facilitating contact with people; the community's close relationship with local flora resources; maintenance of homegardens, which are very productive; and being located in a mountainous environment, a socioecological context little studied in Brazil.

### Ethnobiological study

Initially, a meeting was held at with members of the Community Residents Association in order to describe the project. Those who agreed to contribute to the study were asked to sign the Informed Consent Form (ICF). This study was submitted to and approved by the ethics committee for research with human beings at the Federal University of Vale do São Francisco (CAAE: 80,902,217.2.0000.5196). Also, it was obtained consent to access traditional knowledge with the National Genetic Heritage Management System (SISGEN: AE16D85). Data collection took place between May and October 2019.

In the first moment of the interview, socioeconomic data were collected, containing questions such as age, gender, time of residence in the locality and monthly income. In the second moment, the free list technique [17] was applied with the adult(s) present at the residence at the time of the visit, which we call “head of household.” It is noteworthy that only interviewees over 18 years of age were interviewed. At this stage, participants were asked to list the food plants known and/or used by them. Researchers recorded each plant in exactly the order it was mentioned. After citing the last recalled plant, we proceeded with the non-specific induction technique; it was made as the “new reading” technique to stimulate the recall of other plants [18]. From the list provided by each person, researchers then asked the following questions about the plants mentioned: Which part is used? What form of consumption do you know? What are the places where food plants are collected? Why this location? What is the collection frequency? Have you ever consumed this plant? When did you last consume this plant? Is this food plant commercialized? Is there another use for this plant? If so which? What part of the plant is used for this new use? Are there any plants that you no longer consume?

It is important to note that the collection sites were also categorized into: (1) homegardens: anthropized environments, located near one’s residence, in which domestic animals and plants (used mainly for food, medicinal and ornamental purposes) are found; (2) the swidden: an area of territorial extension in which agricultural activities are developed, for example: intercropping of corn and beans; (3) the vegetation areas: are areas apparently little anthropized which are be located in low areas, on the slopes and/or at the top of the mountains?

### Data analysis

After general questions about the uses and possession of free lists, the preference of the plants was analyzed based on the salience index (SI), which considered the frequency of citation and the average position of the items in the lists obtained [17]. The protocol proposed by Chaves, Nascimento and Albuquerque [19] was applied, which allowed the verification of significantly more prominent plants. Thus, the plants were divided into three groups: (1) plants that obtained significantly high salience values and were distinct from the null scenario; (2) plants whose observed values did not differ from the null model; and (3) plants with the lowest salience values, which differ significantly from the null model. These analyses were performed using the software R version 4.0.5 [20]. For graphical representation of uses, a string diagram was applied using the “ethnoChord” function of the “EthnobotanyR” package. The research was based on the citation of use; in this sense, a few plants mentioned do not occur in the region, such as Araucaria angustifolia, “xixó” and “jacuti,” or are not cultivated, all of which are obtained in markets. For most plants, among the exotic ones, such as citrus, banana, and avocado, and native species, umbu, caroá, licuri, among others, the identification was carried out in situ by authors who have a background in botany. Finally, for some plants of spontaneous occurrence, unknown to the authors, and those that generated doubts in the identification, the material was collected and taken for identification by specialists linked to the herbarium of NEMA (Núcleo de Ecologia e Monitoramento Ambiental)/UNIVASF. It is noteworthy that all analyses were performed based on the quantity of food plants and forms of use mentioned locally. In this sense, as in the cases of Jaca and Jaca pirão, as well as Anoma and Pinha, which are the species *Artocarpus heterophyllus* Lam. (Moraceae) and *Annona squamosa* L. (Annonaceae), respectively, the diversity perceived by people was considered, and therefore, four plants instead of two.

In order to assess the influence of socioeconomic factors on the knowledge, we used the number of citations of the food plants and forms of consumption per people. For these analyses, we follow the protocol suggested by Finch and Finch [21] GLM lasso, but to calculate the value of “p,” the lassopv function of the “lassopv” package was used. The Poisson distribution was used. The explanatory (independent) variables were: time of residence, family income, gender, preferred place of collection (vegetated area close to the village and areas of high anthropic intensity, such as homegardens, being binary variables, 1 for vegetation area and 2 for anthropogenic areas), retired (binary variable, retired 1 and not retired 0) and number of residents in the house. There were two response (dependent) variables: the number of food plants and forms of consumption cited. All variables were also standardized prior to the analyses. The analyses were performed using the software R 4.0.5 [20], using packages “scales,” “glmnet” and “lassopv.”

## Results

### Diversity of known and used food plants

The village of Serra dos Morgados, according to people's accounts, was founded by the Morgado family, the first to live in Serra do Baixio, at the end of the nineteenth century. In this region, the “serras” (mountains) are called “grotas” and its residents call themselves “groteiros,” in a clear differentiation from the “caatingueiros,” people who live in the driest areas of the municipality. It is noteworthy that the mountains are places of occurrence of many springs and rivers, as well as freshwater groundwater, which supply almost the entire region, including the residents of the two communities (Morgados and Berinjela). As a result of climate change, deforestation, excessive drilling of wells, mining activities and, more recently, the implementation of wind farms, some of these springs and important rivers and waterfalls have dried up, making the water issue a central issue in the region. The SM community is typically rural, whose main activity is organic family farming and small livestock. According to reports from the inhabitants of the mountains, agriculture has always been the main activity in the community, especially the cultivation of beans; corn; manioc, there were four flour mills in the community; and coffee. In the past, red rice (*Oryza sativa*) was also cultivated, grown in the swamps, and sugarcane (*Saccharum officinarum*), with the associated production of “rapadura” (sweet). According to people, these plantations were located in steep areas, far from the banks of the Estiva River. This was very important for the whole community, but due to the drilling of wells and construction of cisterns, following recommendations from government agencies, the “Estiva” river is “dead,” dry, no longer serving the community as in past times. Recently, some community members are slowly resuming coffee planting.

**Fig. 2:**
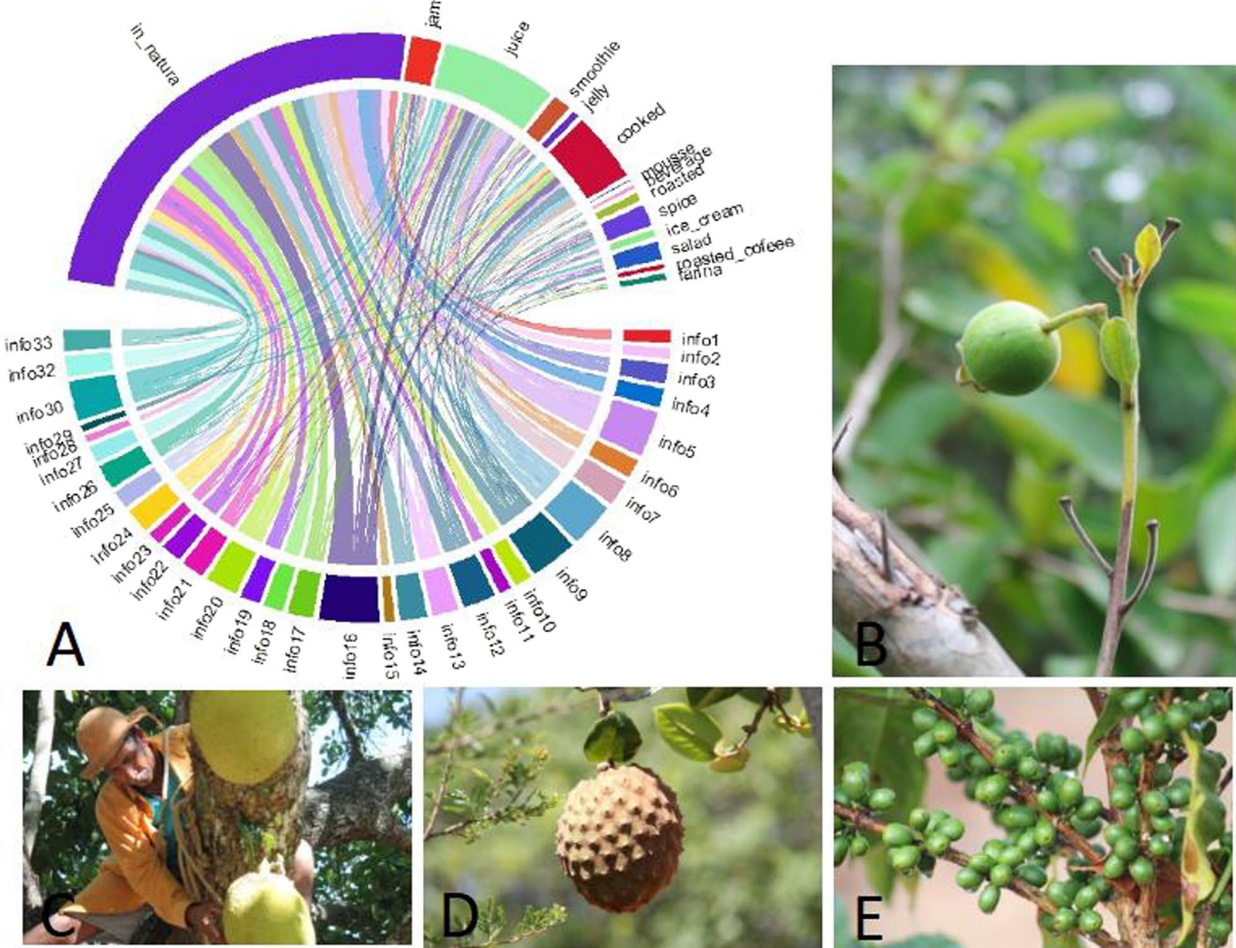
Chord diagram (**A**) represented the distribution of use form of the edible plants per people in Serra dos Morgados. Examples of some of the plants mentioned: “Cheirosa”—*Psidium ganevii.* (**B**), “Jaca”—*Artocarpus heterophyllus* Lam. (**C**), “Bruto da Grota”—*Annona coriacea* Mart. (**D**) and coffee—*Coffea arabica* L. (**E**). Authors

In the community of 100 families, 33 families, one people per family, called householder, were interviewed, being 8 men and 25 women (Table 1). Regarding the known plant diversity, people cited 98 species of food plants; of these, 38 were identified as being of spontaneous occurrence. The average number of plants cited by men and women was approximately 23 and 19, respectively. Most food plants in the community have the fruit as the preferential part used, especially for fresh consumption (79.8% of respondents), followed by juice (18.12%), cooked (9.97%), sweets (3.78%) and spices (3.47%) (Fig. 2).

**Table 1:** Socioeconomic information

Socioeconomic category	Class	Number of informants (%)
Gender	Female	25 (76%)
	Male	8 (24%)
Age	31–40	1 (3%)
	51–60	2 (6%)
	> 60	30 (91%)
Education	Primary school I	21 (63%)
	Primary school II	8 (25%)
	Higher education	2 (6%)
	Illiterates	2 (6%)
Occupation	Retired	19 (56%)
	Farms	7 (22%)
	Housemaids	4 (13%)
	Salaried	3 (9%)
Time in the community	Since childhood	21 (62.5%)
	Lived, for some time, in other places	12 (38.5%)
Monthly income (individual)^a^	Up to a minimum wage	25 (76%)
	Up to two minimum wages	8 (24%)
Monthly income (home)^a^	Up to a minimum wage	15 (45%)
	Up to two minimum wages	13 (39%)
	Above two minimum wages	5 (16%)
Government assistance	Receive	6 (19%)
	No receive	27 (81%)

^a^The minimum wage in Brazil on 10/18/2020 is R$ 1045.00 (US$ 184,96)

The collection of plants takes place mainly in anthropic areas (56.74% of citations), such as in a homegarden or in “swidden areas,” followed by areas of vegetation located near the community (37.32% of citations), both areas (anthropic and vegetation) (4.64% of citations), and purchased at markets and street markets (1.29% of citations). Among the plants found in homegardens, the following stand out: abacate (*Persea americana* Mill.) (50% of citations), goiaba (*Psidium guajava* L.) (46.9%), banana (*Musa paradisiaca* L.) (43.7%), jaca (*Artocarpus heterophyllus* Lam.) (43.7%), manga (*Mangifera indica* L.) (37.5%), laranja (*Citrus* sp.) (34.4%) and coffee (*Coffea arabica* L.) (31.25%). In homegardens, 59.4% of respondents market the resources that are managed in these areas in addition to direct use for consumption. The sale of these products takes place in the community itself, as well as in the local market in the main town, Jaguarari.

### Analysis of cultural salience (CS) of food plants of Serra dos Morgados

Based on the salience analysis of the free lists (Table 2), the following species stand out among those with the highest salience indices (SI): “cheirosa” (*Psidium ganevii* Landrum & Funch) (SI = 0.5683) (cited by 90.9% of people), followed by “jaca” (*Artocarpus heterophyllus* Lam.) (SI = 0.4611) (cited by 60.6% of people); “massaranduba” (*Micropholis sp.*) (SI = 0.4319) (cited by 99% of people); “abacate” (*Persea americana* L.) (SI = 0.3962) (cited by 57.6% of people); “laranja” (*Citrus sp.*) (SI = 0.3554) (cited by 51.5% of people); “manga” (*Mangifera indica* L.) (SI = 0.3440) (cited by 48.5% of people); banana (*Musa paradisiaca* L.) (SI = 0.3338) (cited by 51.5% of people); “araça” (*Campomanesia guazumifolia* (Cambess.) O. Berg) (SI = 0.3316) (cited by 54.5% of people); “goiaba” (*Psidium guajava* L.) (SI = 0.3202) (cited by 54.5% of people); and “cambuí” (*Siphoneugena sp.*) (SI = 0.3137) (cited by 48.5% of people), emphasizing that all are fruit trees, consumed mainly fresh (Table 2). The most prominent family, in relation to the number of species, is the family Myrtaceae. Spontaneously occurring plants are collected in areas of vegetation and fields, among which the most frequently mentioned were “araça” (*C. guazumifolia*) (SI = 0.3316), “umbuzeiro” (*Spondias tuberosa* L.) (SI = 0.1630) (cited by 30.3% of people) and “maracujá de boi” (*Passiflora cincinnata* Mast.) (SI = 0.1579) (cited by 36.4% of people).

**Table 2:** Food plants mentioned by people from the Serra dos Morgados Village, Bahia Northeastern Brazil

Common name	Scientific name (Família)	Consumed part	Form of consumed	SI^a^ (*P* value)
Abacate	*Persea americana* Mill. (Lauraceae)	Fruit	In natura/smoothie	0.3959 (0.0000)
Abacaxi	*Ananas comosus* (L.) Merr. (Bromeliaceae)	Fruit	In natura/juice	0.0979 (0.4672)
Abóbora	*Cucurbita sp.* (Cucurbitaceae)	Fruit	Cooked	0.0731 (0.2459)
Acerola	*Malpighia emarginata* DC. (Malpighiaceae)	Fruit	In natura	0.1399 (0.2030)
Alface	*Lactuca sativa* L. (Asteraceae)	Leaf	Salad	0.1210 (0.3327)
Alho poro	*Allium ampeloprasum* L. (Amaryllidaceae)	Leaf (“Stalk”)	Spice	0.0163 (0.0067)
Amora	*Rubus sp.* (Rosaceae)	Fruit	In natura	0.0416 (0.0587)
Andu	*Cajanus cajan* (L.) Huth (Fabaceae)	Fruit	Cooked	0.0424 (0.0618)
Anoma	*Annona squamosa* L. (Annonaceae)	Fruit	In natura/juice	0.0035 (0.0006)
Araçá	*Campomanesia guazumifolia* (Cambess.) O. Berg (Myrtaceae)	Fruit	In natura	0.3316 (0.0000)
Araucária	*Araucaria angustifolia* (Bert.) O. Kuntze (Araucaraceae)	Fruit/seeds	Cooked/roasted	0.0052 (0.0009)
Banana	*Musa paradisiaca* L. (Musaceae)	Fruit	In natura/smoothie/roasted	0.3339 (0.0000)
Batata doce	*Ipomoea batatas* (L.) Lam (Convolvulaceae)	Root	Cooked/roasted	0.0820 (0.3212)
Beterraba	*Beta vulgaris* L. (Quenopodiaceae)	Root	In natura/cooked	0.0983 (0.4703)
Bredo	*Amaranthus viridis* L. (Amaranthaceae)	Leaf	Cooked/in natura	0.0230 (0.0141)
Bruto da grota	*Annona coriacea* Mart. *(Annonaceae)*	Fruit	In natura	0.2567 (0.0016)
Budinho	*Amaranthus spinosus* L. (Amaranthaceae)	Fruit	In natura	0.0959 (0.4368)
Burra leiteira	*Euphorbia hyssopifolia* L. (Euphorbiaceae)	Leaf	Cooked	0.0015 (0.0001)
Café	*Coffea arabica *L. (Rubiaceae)	Fruit	Roasted	0.1438 (0.1608)
Cajá	*Spondias mombin* L. (Anacardiaceae)	Fruit	In natura/juice	0.0447 (0.0676)
Caju	*Anacardium occidentale* L. (Anacardiaceae)	Pseudofruit (“Chestnut”)	In natura/juice/roasted	0.1403 (0.2054)
Caju do mato	*Anacardium sp.* (Anacardiaceae)	Fruit	In natura	0.0199 (0.0091)
Cambucá	*Eugenia sp.* (Myrtaceae)	Fruit	In natura	0.2049 (0.0196)
Cambuí	*Siphoneugena sp.* (Myrtaceae)	Fruit	In natura	0.3142 (0.0001)
Cana de macaco	*Costus sp.* (Costaceae)	Stalk	In natura	0.0430 (0.0615)
Carambola	*Averrhoa carambola* L. (Oxalidaceae)	Fruit	In natura	0.0268 (0.0197)
Caroá	*Neoglaziovia variegata* (Arruda) Mez (Bromeliaceae)	Fruit	In natura/cooked/roasted	0.1226 (0.3203)
Cebolinha	*Allium schoenoprasum* L. (Amaryllidaceae)	Leaf	Spice	0.0373 (0.0449)
Cenoura	*Daucus carota* L. (Apiaceae)	Root	In natura/cooked/crumbs	0.0935 (0.4251)
Cheirosa	*Psidium ganevii* Landrum & Funch (Myrtaceae)	Fruit	In natura	0.5683 (0.0000)
Chuchu	*Sechium edule* (Jacq.) Sw. (Cucurbitaceae)	Fruit	Cooked	0.0357 (0.0396)
Coco	*Cocos nucifera* L. (Arecaceae)	Fruit	In natura	0.0060 (0.0013)
Coentro	*Coriandrum sativum* L. (Apiaceae)	Leaf	Spice	0.0855 (0.3515)
Condessa	*Annona reticulata* L. (Annonaceae)	Fruit	In natura	0.0479 (0.0844)
Couve	*Brassica sp.* (Brassicaceae)	Leaf	Salad/cooked	0.0844 (0.3417)
Feijão	*Phaseolus vulgaris* L. (Fabaceae)	Fruit	Cooked	0.0663 (0.1941)
Genipapo	*Genipa americana* L. (Rubiaceae)	Fruit	Juice/liquor	0.0046 (0.0009)
Goiaba	*Psidium guajava* L. (Myrtaceae)	Fruit	In natura	0.3206 (0.0000)
Graviola	*Annona muricata* L. (Annonaceae)	Fruit	In natura	0.1435 (0.1818)
Guabiraba	*Campomanesia xanthocarpa* Mart. ex O. Berg (Myrtaceae)	Fruit	In natura	0.2317 (0.0054)
Inhame	*Dioscorea sp.* (Dioscoreaceae)	Root	Cooked	0.0312 (0.0275)
Ingá	*Inga sp.* (Fabaceae)	Fruit	In natura	0.2619 (0.0011)
Jabuticaba	*Plinia cauliflora* (DC.) Kausel (Myrtaceae)	Fruit	In natura	0.2401 (0.0023)
Jaca	*Artocarpus heterophyllus* Lam. (Moraceae)	Fruit and seed	In natura/cooked	0.4610 (0.0000)
Jaca pirão	Artocarpus heterophyllus Lam. (Moraceae)	Fruit	In natura	0.0208 (0.0101)
Jacuti	Indeterminate	Fruit	In natura	0.0134 (0.0043)
Jambolão	*Syzygium cumini* (L.) Skeels (Myrtaceae)	Leaf	Cooked	0.0179 (0.0071)
Jatobá	*Hymenaea sp.* (Fabaceae)	Fruit	In natura	0.0282 (0.0214)
João gomes	*Talinum paniculatum* (Jacq.) Gaertn. (Talinaceae)	Leaf	Cooked	0.0333 (0.0331)
Juazeiro	*Ziziphus* joazeiro Mart. (Rhamnaceae)	Fruit	In natura	0.0501 (0.0913)
Laranja	*Citrus sp.* (Rutaceae)	Fruit	In natura	0.3557 (0.0000)
Licuri	*Syagrus coronata* (Mart.) Becc. (Arecaceae)	Fruit	In natura	0.1465 (0.1720)
Lima	*Citrus limettioides* Tanaka (Rutaceae)	Fruit	In natura	0.0253 (0.0160)
Limão	*Citrus limonum* Risso (Rutaceae)	Fruit	In natura/juice	0.1079 (0.4540)
Macambira	*Bromelia laciniosa* Mart. ex Schult. f. (Bromeliaceae)	Fruit	In natura	0.0203 (0.0095)
Mamão	*Carica papaya* L. (Caricaceae)	Fruit	In natura	0.1109 (0.4270)
Mamão de veado	*Solanum sp.* (Solanaceae)	Fruit	In natura	0.0013 (0.0000)
Mandacaru	*Cereus jamacaru* DC. (Cactaceae)	Fruit	In natura	0.0748 (0.2516)
Mandioca	*Manihot esculenta* Crantz (Euphorbiaceae)	Root	Root/flour	0.1260 (0.3037)
Manga	*Mangifera indica* L. (Anacardiaceae)	Fruit	In natura/juice	0.3438 (0.0000)
Maracujá_de_boi	*Passiflora cincinnata* Mast. (Passifloraceae)	Fruit	Juice	0.1579 (0.1190)
Maracujá_doce	*Passiflora sp.* (Passifloraceae)	Fruit	In natura/Juice	0.1964 (0.0282)
Maracujina	*Passiflora edulis* Sims (Passifloraceae)	Fruit	Juice	0.0403 (0.0517)
Massaranduba	*Micropholis sp.* (Sapotaceae)	Fruit	In natura	0.4326 (0.0000)
Maxixe	*Cucumis anguria* L. (Cucurbitaceae)	Fruit	Cooked	0.0313 (0.0280)
Melancia	*Citrullus lanatus* (Thunb.) Mansf. (Cucurbitaceae)	Fruit	In natura	0.0914 (0.3967)
Melão	*Cucumis melo* L. (Cucurbitaceae)	Fruit	In natura/juice	0.0139 (0.0044)
Milho	*Zea mays* L. (Poaceae)	Seed (“grain”)	In natura/cooked	0.0503 (0.0921)
Murici	*Byrsonima sericea DC.* (Malpighiaceae)	Fruit	In natura	0.0780 (0.2781)
Murta	*Myrtus communis* L. (Myrtaceae)	Fruit	In natura	0.0592 (0.1413)
Oiti	*Licania tomentosa* (Benth.) Fritsch (Chrysobalanaceae)	Fruit	In natura	0.2788 (0.0005)
Olho de porco	*Cordiera sp.* (Rubiaceae)	Fruit	In natura/Juice	0.1039 (0.5086)
Palma	*Opuntia ficus-indica* (L.) Mill. (Cactaceae)	Fruit and Cladodium	In natura/cooked	0.0760 (0.2613)
Pepino	*Cucumis sativus* L. (Cucurbitaceae)	Fruit	In natura/cooked	0.0313 (0.0280)
Pimenta	*Capsicum sp.* (Solanaceae)	Fruit	Spice	0.0199 (0.0091)
Pinha	*Annona squamosa L.* (Annonaceae)	Fruit	In natura	0.1348 (0.2413)
Pinha braba	*Annona sp.* (Annonaceae)	Fruit	In natura	0.0208 (0.0101)
Pitanga	*Eugenia uniflora* L. (Myrtaceae)	Fruit	In natura	0.0790 (0.2873)
Pitomba	*Talisia esculenta* (A. St-Hil.) Radlik. (Sapindaceae)	Fruit	In natura	0.0195 (0.0087)
Pitomba de cágado	*Myrciaria sp.* (Myrtaceae)	Fruit	In natura	0.0156 (0.0054)
Pursá	*Myrcia splendens* (Sw.) DC (Myrtaceae)	Fruit	In natura/juice	0.2640 (0.0011)
Quiabo	*Abelmoschus esculentus* (L.) Moench (Malvaceae)	Fruit	Cooked	0.0052 (0.0010)
Repolho	*Brassica sp.*(Brassicaceae)	Leaf	Salad/cooked	0.0170 (0.0063)
Salsa	*Petroselinum crispum* (Mill.) Mansf. (Apiaceae)	Leaf	Spice	0.0402 (0.0512)
Seriguela	*Spondias purpurea* L. (Anacardiaceae)	Fruit	In natura/juice/jam	0.1878 (0.0397)
Sapucaia	*Bowdichia virgilioides* Kunth (Fabaceae)	Fruit	In natura	0.0781 (0.2792)
Serralha	*Sonchus oleraceus* L. (Asteraceae)	Leaf	Cooked	0.0304 (0.0260)
Taioba	*Xanthosoma sagittifolium* K. Koch (Araceae)	Leaf	Spice/flour	0.0617 (0.1574)
Tamarindo	*Tamarindus indica* L. (Fabaceae)	Fruit	In natura/juice	0.0565 (0.1247)
Tamoia	*Annona vepretorum* Mart. (Annonaceae)	Fruit	In natura	0.0038 (0.0009)
Tangerina	*Citrus reticulata* Blanco (Rutaceae)	Fruit	In natura	0.2766 (0.0005)
Tomate	*Solanum lycopersicum* Lam. (Solanaceae)	Fruit	In natura	0.0104 (0.0029)
Tomate cereja	*Solanum lycopersicum var. cerasiforme* (Alef.) Voss (Solanaceae)	Fruit	In natura	0.0273 (0.0197)
Umbuzeiro	*Spondias tuberosa* L. (Anacardiaceae)	Fruit	In natura	0.1639 (0.0953)
Urucum	*Bixa orellana* L. (Bixaceae)	Seed	Coloring	0.0089 (0.0025)
Xique xique	*Pilocereus gounellei* F.A.C. Weber (Cactaceae)	Fruit	In natura	0.0104 (0.0029)
Xixó	Indeterminate	Fruit	In natura/juice	0.0069 (0.0017)

Regarding the lowest indexes, all these plants had only 3% of citations, which also differ significantly from the null model; we observed “mamão de veado” (*Solanum sp.*) (SI = 0.0013); burra leiteira (*Euphorbia hyssopifolia* L.) (SI = 0.0015); “anomá” (*Annona squamosa* L.) (SI = 0.0035); “tamóia” (*Annona vepretorum* Mart.) (SI = 0.0038); “genipapo” (*Genipa americana* L.) (SI = 0.0046); “araucária” (*Araucaria angustifolia* (Bert.) O. Kuntze) (SI = 0.0052); “quiabo” (*Abelmoschus esculentus* (L.) Moench) (SI = 0.0052); and “coco” (*Cocos nucifera* L.) (SI = 0.0058) (Table 2).

### Influence of socioeconomic variables

Among the socioeconomic variables evaluated (Table 3), only family income significantly influenced knowledge of food plants. From this analysis, it was possible to verify that people with higher family income are the ones who cited a greater number of plants (Table 3). When socioeconomic variables were analyzed considering the forms of use of food plants, the explanatory model suffered a small variation, in which family income continued to be the significant variable; however, the variable preferred location for collection, homegarden, was added to the model, although it did not receive significance (Table 3).

**Table 3:** Standardized Model Coefficients for the predictors of knowledge and forms of consumed

Knowledge (number of citations)	
(Intercept)	15.190262372
Gender	NA
Age	NA
Time of residence	NA
Family income	0.003915268^a^
Preferred place of collection	NA
Retired	NA
Forms of consumed	
(Intercept)	21.359610309
Gender	NA
Age	NA
Time of residence	NA
Family income	0.009304561^a^
Preferred place of collection	− 3.489586219^b^
Retired	NA

NA = Variable not selected for inclusion in the final model

^a^Statistically significant at alfa = 0.05

^b^Variable selected, but not significant at alfa = 0.05

## Discussion

### Knowledge, use and preference of food plants

The predominance of species belonging to the Myrtaceae family reflects a trend in studies of food plants development in Brazil [23]. This family groups together a number of relevant food species, in which the most consumed part is the fruit, with attractive organoleptic characteristics, such as smell, flavor and shape. Also, the fact that the fruits were the most commercialized part of the plant may be related to two issues that are worth mentioning: 1) part of the plant that, in most forms of consumption, requires little preparation time; and 2) the community plays an important role for the nearby municipality, with regard to the supply of fruit for sale. Different studies have found a similar trend, especially in places where the species are consumed and traded [10, 26]. As it is a widely distributed family and with representatives that group important characteristics, such as those described above, a significant number of species linked to this family were expected.

The maintenance of homegardens in the Serra dos Morgados community may have influenced the people to quickly remember the plants present in the area. Many studies on food plants, linked to the maintenance of homegardens, concluded that people prioritize keeping plants close to their homes that can be used daily [23]. The fact that food plants are commercialized also deserves attention, as these plants have an important economic value, which can influence the frequency of citation of certain species and their cultural importance, even if most of these plants are exotic. The predominant presence of exotic plants needs to be considered, as it raises some important issues, such as the use of memory to store information considered useful; greater cultural acceptance of exotic plants by people in the municipality of Jaguarari, since several families in the community sell these species in the urban center and, finally, the ease of maintenance of these species close to their homes. However, it is important to highlight that not all species that are commercialized are cultivated by local communities. Many of the resources may come from different regions of the country, as is the case with Araucaria.

In this way, suppose that certain plant species, which are kept close to the residences, are those that add both an economic value and a positive cost–benefit relationship, with regard to collection and use; all this can justify the maintenance of greater number of exotic species in backyards. On the other hand, in studies carried out in arid and semiarid regions, many native species used for food are still found, especially in places where there is a shortage of other resources, both food and financial [5–9].

From the above, it called our attention to the fact that we have a native species which presented the highest value of cultural importance; it is *P. gannevii*, popularly known as “cheirosa.” This is an endemic species for the northeast region, commonly found in areas from 800 to 1200 m of altitude, especially in the Bahia portion of the “cadeia do espinhaço” (mountain chain extending through the states of Minas Gerais and Bahia) [24, 25]. This plant has a restricted occurrence, what is important to describe the relationship between people in the Serra dos Morgados community and this species. It is noteworthy that before this study there was no record of occurrence of this species in the region, reinforcing the importance of this study for ethnoprospecting food plants. Finally, it is interesting to say that the “cheirosa” plant was considered the species with the most widespread knowledge in the community, being found everywhere, especially in homegardens. Thus, it is important to emphasize that, among so many exotic plants, there is a native species with considerable local prominence, which leads us to suggest that plants of spontaneous occurrence, but which have a high nutritional and cultural value, linked to practices can be maintained over time in intercropping with exotic species of equal nutritional and economic importance to these populations. A similar result was found in the Adi region of India, where the authors suggested that those culturally important species are more likely to be maintained and used by local communities [15].

Campos et al. [26] highlight a similar behavior of communities that maintain homegardens with the presence of spontaneously occurring plants and exotic plants. In the same study, Campos et al. [26] studied the three communities; one of them that maintained a closer relationship with the homegardens showed less knowledge and use of spontaneously occurring plants, leading to the conclusion that homegardens interfered with the knowledge and use about native species. On the other hand, recently Albuquerque et al. [27] proposed the theory of resource maximization, in which they postulate that the structuring and organization of socioecological systems are guided by the human behavior of maximizing benefits and reducing costs. In this sense, considering that for the people of Serra dos Morgados, the main areas for obtaining food resources are anthropized environments, making them very productive for the population of the region, not only for domestic consumption, but for commercial use.

However, such evidence found here supports the need to understand why, in view of a great biodiversity and regional food potential, consumption is still restricted to a small number of food plants. In this sense, studies on this understanding should be expanded, also enabling the prospecting of food plants, an aspect that will greatly contribute to food security [8]. Another explanation that deserves to be mentioned regarding the lesser use of native resources that can be used for food, especially in times of scarcity, is the fact that residents receive financial aid from the federal government. Thus, it is suggested that the improvement in financial conditions and access to certain foods can lead to a reduction in the collection of native plant resources; the factor is also observed in other studies, with the same theme, carried out in Brazil in previous years [5, 26, 28, 29].

### Influence of socioeconomic variables

In the Serra dos Morgados, neither the gender nor the time of residence influenced the quantity of known plants and forms of local consumption. In this sense, Ojelel and Kakudidi [30] studying the Obalanga Community found that knowledge about food plants is directly related to the functions performed by an individual in the community, in Uganda, Africa. In this sense, it is important to highlight that, in Serra dos Morgados, both men and women manage the homegardens. In addition, there is no single person responsible for managing these plants, which may explain the fact that they are equally aware of this resource. In most studies that find differences in knowledge between genders, the authors explain such differences by relating them to their social function within communities [4, 31], suggesting that the distribution of knowledge between men and women is dynamic and needs to be considered based on particular socioecological structures and/or specific practices, developed at the study sites.

The family's income had a positive relationship with the number of plants mentioned and forms of consumption. Considering homegardens as productive areas and the fact that the majority of respondents were retired, the income factor varied positively with the dependent variables analyzed. The most common scenario of studies that analyzed the influence of income on knowledge was the opposite of that observed in Serra dos Morgados. As Medeiros et al. [32] points out, low-income families would tend to relate more closely to natural resources, considering them for their subsistence. In this sense, the greater the knowledge about natural resources, the lower the people's income would be, indicating that in the first moment the income would be influencing in the opposite direction in the quantity of resource used [32]. However, this interference of the income factor in the uses of resources, warns Medeiros et al. [32], may be representing not what is earned, but savings on domestic expenses.

## Conclusion

The homegardens of Serra dos Morgados are spaces of great manipulation of food plants, especially fruit trees. This finding is justified, mainly because these environments are managed to meet local demands for domestic and commercial use. This continuous stimulus may also be responsible for the immediate memory of these plants [33], evidenced in the high salience rates of fruit trees, and found mainly in these environments (homegardens). It is noteworthy that the entire spatial context of homegardens is fundamental in the development of autonomic memory associated with the use of food plants [33–35]. In any case, this study contributes to the understanding of the dynamics of knowledge and use of fruit food plants in regions that have a high dependence on the managed environments, which may have important implications for strategies for managing environments and local knowledge. Regarding the influence of socioeconomic variables, it was possible to observe that the socioecological context in which the study was developed (dynamics of collection and use of resources) significantly influenced the roles performed by men and women, as well as family income.

We believe that when considering the potential for studies on patterns of use of food species, this study is of great relevance to drive further research on the nutritional potential of species, as well as studies of ethnoculinary, as suggested by Jacob et al. [8].

## Data Availability

Please contact author for data requests.
